# Male meiosis, morphometric analysis and distribution pattern of 2× and 4× cytotypes of *Ranunculus hirtellus* Royle, 1834 (Ranunculaceae) from the cold regions of northwest Himalayas (India)

**DOI:** 10.3897/CompCytogen.v5i3.1359

**Published:** 2011-08-24

**Authors:** Puneet Kumar, Vijay Kumar Singhal

**Affiliations:** Department of Botany, Punjabi University, Patiala -147 002, Punjab, India

**Keywords:** chromosome number, cytotype, cytomixis, Lahaul-Spiti, Manali hills, Manimahesh hills, meiotic abnormalities, stomata

## Abstract

In this study, we examined the chromosome number, detailed male meiosis, microsporogenesis, pollen fertility and morphological features and distribution of 2× and 4× cytotypes of *Ranunculus hirtellus* Royle, 1834. The majority of the populations scored now from cold regions of the northwest Himalayas showed tetraploid (n=16) meiotic chromosome count and one of the populations studied from the Manimahesh hills existed at diploid level (n=8). The individuals of diploid cytotype exhibited perfectly normal meiotic course resulting in 100% pollen fertility and pollen grains of uniform sizes. On the other hand, the plants of the tetraploid cytotype from all the populations in spite of showing normal bivalent formation and equal distribution to the opposite poles at anaphases showed various meiotic abnormalities. The most prominent among these meiotic abnormalities was the cytomixis which involved inter PMC (pollen mother cell) chromatin material transfer at different stages of meiosis-I. The phenomenon of cytomixis induced various meiotic abnormalities which include chromatin stickiness, pycnotic chromatin, laggards and chromatin bridges, out of plate bivalents at metaphase-I, disoriented chromatin material at anaphase/telophase and micronuclei. Consequently, these populations exhibited varying percentages of pollen sterility (24 - 77 %) and pollen grains of heterogeneous sizes. Analysis of various morphometric features including the stomata in 2× and 4× cytotypes showed that increase in ploidy level in the species is correlated with gigantism of vegetative and floral characters and the two cytotypes can be distinguished from each other on the basis of morphological characters. The distribution patterns of the 2× and 4× cytotypes now detected and 2×, 3×, 4× cytotypes detected earlier by workers from other regions of the Indian Himalayas have also been discussed.

## Introduction

*Ranunculus hirtellus* Royle, 1834 (Ranunculaceae), a perennial erect or decumbent herb, distinctly pubescent with fibrous and shortly fusiform root stock is endemic to Himalayas, and distributed in the temperate, sub-alpine and alpine slopes in North-West to North-East Himalaya, temperate to subalpine slopes at 2000 – 4500 m in the states of Jammu & Kashmir, Himachal Pradesh, Uttarakhand, Sikkim, and Arunachal Pradesh and also in Afghanistan, Pakistan, Nepal and Tibet (Sharma et al. 1993, Srivastava 2010). The species is medicinally important as the paste made by crushing roots in cow’s urine is used to cure the swellings of testes by the tribal communities of Chhota Bhangal area of Kangra district in Himachal Pradesh (Uniyal et al. 2006). Besides, the species is also used to cure skin diseases and wounds, and as a vermifacient, cooling agent and anthelmintic in other parts of the Himalayas in India (Jain 1991, Iyer 1992, Kumar 2010, Pharswan et al. 2010).

The species is highly variable with respect to habit, plant size, shape and hairiness of leaves and sepals, hairiness of pedicels, and size of flowers (Aswal and Mehrotra 1994). The information gathered from various Indexes to Plant Chromosome Numbers (Goldblatt 1981, 1984, 1985, 1988, Kumar and Subramanian 1986, Khatoon and Ali 1993) also revealed that the species is equally variable in terms of chromosome number (2n=14, 16, 24, 28, 32) and level of ploidy (2×, 3×, 4×). Earlier chromosomal studies in the species were confined to merely counting the chromosome number and no attempt has been made to correlate the extent of morphological variability among different cytotypes and their relative distribution patterns in the Himalayas.

Our research group has been engaged in the study of cytological aspects of plants of cold deserts of India through male meiosis since 2006. Male meiosis of more than 300 species from the cold desert areas of Chamba, Lahaul-Spiti and Kinnaur districts of Himachal Pradesh has been studied and various aberrations were detected during male meiosis in *Caltha palustris* Linnaeus, 1753(Kumar and Singhal 2008), *Meconopsis aculeata* Royle, 1834(Singhal and Kumar 2008a), *Hippophae rhamnoides* Linnaeus, 1753(Singhal et al. 2008), *Papaver dubium* Linnaeus, 1753 *(*Singhal and Kaur 2009), *Anemone rivularis* Buch.-Ham. ex DC., 1817(Singhal et al. 2009a), *Inula cuspidata* (DC.) C. B. Clarke, 1876 (Kaur et al. 2010), *Clematis orientalis* Linnaeus, 1753(Kumar et al. 2010), *Ranunculus laetus* Wall. ex Royle, 1839 (Kumar et al. 2011), *Thalictrum foetidum* Linnaeus, 1753(Singhal et al. 2011), *Dianthus angulatus* Royle ex Benth., 1835 (Kumar et al. in press) and *Lindelofia longiflora* Baill., 1890 (Singhal et al. in press).

The aim of the present research was to study the male meiosis in detail and to find the impact of chromatin transfer in inducing meiotic aberrations and their consequent effect on pollen fertility and pollen size. The purpose of the present study was also to differentiate the 2× and 4× individuals growing wild and also to find out the distribution patterns of different cytotypes in the Indian Himalayas.

## Material and methods

**Figure 1.**
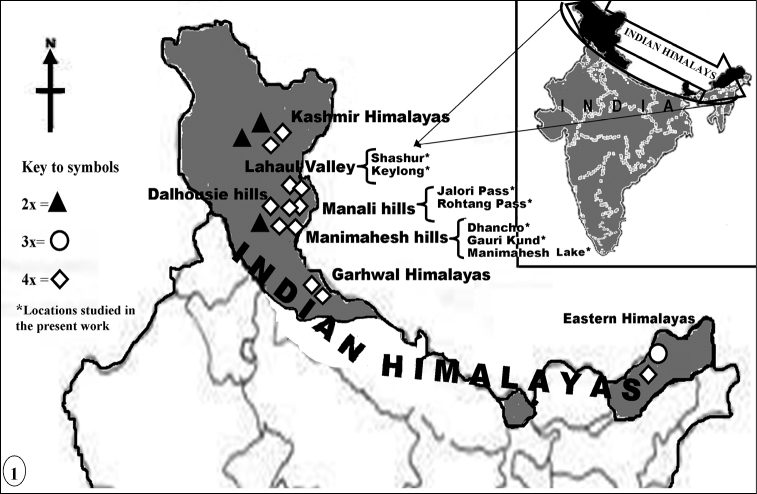
Map showing the distribution pattern of the 2× and 4× cytotypes reported here (marked with asterisks) and the 2×, 3×, 4× cytotypes detected by workers from other regions of the Indian Himalayas.

*Plant material and identification*– Material for male meiotic studies were collected from the wild plants growing in different localities of Lahaul-Spiti, Manimahesh hills and Manali hills of Himachal Pradesh, India in the months of May - July during the years 2008 and 2009 (Table 1, Fig. 1). The identification of the taxon was done by consulting the various floras of the region such as, Flora of Lahaul-Spiti (Aswal and Mehrotra 1994), Flora of Kullu district (Dhaliwal and Sharma 1999) and Flora of Chamba district (Singh and Sharma 2006). Besides, the plant specimens were also compared with the samples in the Herbaria of the Department of Botany, Punjabi University, Patiala (PUN), Botanical Survey of India, Dehra Dun (BSI), and Forest Research Institute, Dehra Dun (FRI). The voucher specimens are deposited in the Herbarium, Department of Botany, Punjabi University, Patiala. The young developing floral buds from healthy plants were fixed in freshly prepared Carnoy’s fixative (6 Ethanol: 3 Chloroform: 1 Glacial acetic acid v; v; v) for 24 hours and subsequently stored in 70% ethanol until analysis.

**Table 1.** List of specimen number/s, meiotic chromosome number, and places of collection with district, province, habitat, latitude and longitude, altitude and habitat of different populations of the diploid (2*n* = 2*×* = 16) and tetraploid (2*n* = 4*×* = 32) cytotypes of *Ranunculus hirtellus*. *Herbarium code as per “Index Herbariorum” by Holmgren and Holmgren (1998).

Cytotype	Specimen number (PUN*)	Meiotic chromosome number (n)	Places of collection with district, province, habitat, latitude and longitude and altitude in meters (Alt. m)
Diploid	51801	8	Gauri Kund, Manimahesh hills, Chamba, Himachal Pradesh, alpine moist slopes, 32°24.11'N; 76°38.25'E , Alt.: 3930 m
Tetraploid	51370	16	Manimahesh Lake, Manimahesh hills, Chamba, Himachal Pradesh, alpine moist slopes, 32°23.91'N; 76°38.30'E , Alt.: 4300 m
	51356	16	Dhancho, Manimahesh hills, Chamba, Himachal Pradesh, along water course, 32°25.18'N; 76°36.53'E , Alt.: 3030 m
	51360	16	Jalori Pass, Manali hills in Kullu, Himachal Pradesh, moist slopes in Oak forest, 31°31.95'N; 77°23.87'E , Alt.: 3140 m
	51364	16	Rohtang Pass, Manali hills in Kullu, Himachal Pradesh, alpine moist slopes, 32°21.84'N; 77°14.59'E , Alt.: 3980 m
	51138	16	Shashur, Lahaul Valley in Lahaul-Spiti, Himachal Pradesh, open and moist grassy slopes among scattered trees of Salix and Juniperus 32°34.56'N; 77°1.54'E , Alt.: 3340 m
	51374	16	Keylong, Lahaul Valley in Lahaul-Spiti, Himachal Pradesh, growing under Salix trees in moist conditions, 32°34.18'N; 77°2.01'E , Alt.: 3340 m

*Chromosome counts and male meiotic analysis*– Developing anthers from floral buds were squashed in 1% acetocarmine and preparations were studied for chromosome counts, and detailed meiotic behavior in pollen mother cells (PMCs) at early prophase-I, metaphase-I (MI), anaphases-I/II (AI/II), telophases-I/II (TI/II) and sporad stage. In populations with normal meiotic course, a total of 10–30 PMCs were examined for determining the chromosome counts while in cytologically abnormal populations 20–50 slides prepared from different anthers/flowers (with 100–200 PMCs) were analyzed in each case.

*Pollen fertility*– Pollen fertility was estimated through stainability tests for which anthers of mature flowers were squashed in glyceroacetocarmine mixture (1:1) or 1% aniline blue dye. 200–500 pollen grains were analyzed in each case for pollen fertility and pollen size. Well-filled pollen grains with uniformly darkly stained cytoplasm were scored as fertile/viable while shrivelled pollen with unstained or poorly stained cytoplasm were counted as sterile/unviable. Pollen fertility was expressed as an average percentage of the stained pollen grains/total pollen grains analyzed. Size of stained pollen grains was measured with occulomicrometer.

*Photomicrographs*– Chromosome spreads were analyzed with Olympus light microscope and the best plates of chromosome counts, meiotic abnormalities, sporads and pollen grains (fertile, sterile) were photographed from the temporary mounts with Nikon Eclipse 80*i* microscope.

## Results

**Figure 2A–D.**
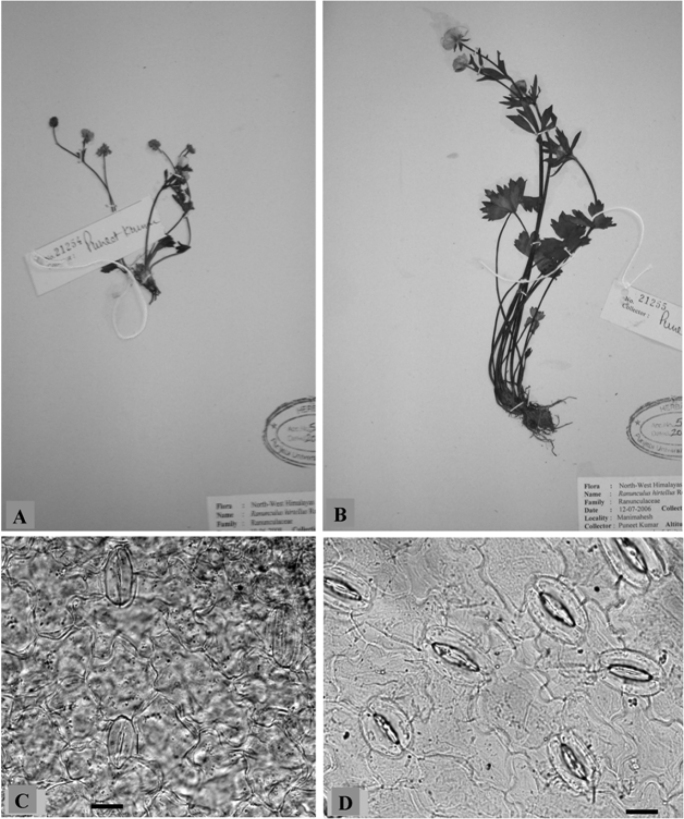
Individuals of *Ranunculus hirtellus* **A** 2× **B** 4× cytotype. Stomata **C** 2× and **D** 4× cytotype. Scale bars = 20 μm.

*Ranunculus hirtellus* has been worked out for male meiosis and morphometric analysis from seven different localities of Manimahesh and Manali hills, and Lahaul-Spiti (Table 1). Two intraspecific cytotypes (Fig. 2, A & B), the diploid (n=8) and the tetraploid (n=16) have been detected in the species. The population scored from the Manimahesh hills was found to be diploid while rest of the six populations studied from Kullu, Chamba and Lahaul-Spiti districts existed at tetraploid level. Cytological and morphometric analysis have been performed on both the cytotypes and data regarding the micro- and macroscopic characters are provided in Table 2.

**Table 2.** Comparison of micro- and macroscopic characters of the diploid (2*n* = 2*×* = 16) and tetraploid (2*n* = 4*×* = 32) cytotypes of *Ranunculus hirtellus*(Figures in the parentheses represent the mean ± standard deviation). *4× populations from Manimahesh Lake and Keylong

S. No.	Characters	Cytotype	
		Diploid	Tetraploid
1.	Meiotic chromosome number (n)	8	16
2.	Plant height (cm)	21.20–23.50 (22.41±0.89)	34.80–37.20 (35.78±0.90)
3.	Radical leaf length (cm)	6.80–10.28 (8.03±1.45)	16.80–22.40 (18.58 ±2.71)
4.	Number of flowers/plant	15–21 (16 ±2.11)	18–27 (25.3±2.7)
5.	Stomatal size (µm)	29.54–39.29 × 17.08–27.10 (34.04±2.47) (21.14±2.56)	34.55–45.46 × 23.56–28.41 (39.15±3.23) (26.98±1.77)
6.	Stomatal density/mm2	63–127	90–137
7.	Stomatal index	11.47	24.94
8.	Pollen grain size (µm)	24.52 - 24.85 × 25.13 - 26.55 (24. 63±0.35) (25.95±1.26)	24.52 × 25.13* 24.85 × 26.55*

### Morphometric analysis

Morphometric analysis involves both macro- and microscopic characters (Table 2). Macroscopic characters including plant height, radical leaf length and number of flowers per plant were studied from all the populations of the tetraploid and one population of the diploid cytotype. The tetraploid plants measured in height were much taller than the diploid. Also the radical leaves were noticed to be much larger in the tetraploid cytotype compared with the diploid. The number of flowers was more in the tetraploid compared to the diploid. Stomata were analysed from 2× population collected from Manimahesh Lake, 4300 mand the 4× population from Keylong, 3340 m (Fig. 2, C & D). The values for stomatal size, density and index were found to be more in the tetraploid compared to the diploid (Table 2). Pollen grains in the diploid cytotype were almost uniform sized whereas in the tetraploid cytotype pollen grains were of variable sizes except for two populations (Table 3).

**Table 3.** Pollen grain size, relative frequency of variable sized pollen grains and pollen sterility in diploid 2*×* and tetraploid 4*×* cytotypes of *Ranunculus hirtellus*(Figures in the parentheses represent the mean ± standard deviation). Rf = relative frequency of variable sized pollen grains.

S. No.	Populations	Pollen grains size (µm)		Rf % age	Pollen sterility % age
		Diploid	Tetraploid		
1.	Gauri Kund	24.52 - 24.85 × 25.13 - 26.55 (24. 63±0.35) (25.95±1.26)		100	00
2.	Dhancho		59.96 × 59.9640.04 × 40.0432.76-36.40 × 29.12 - 36.40(35.25±4.21) (33.72±5.13)21.84 - 29.12 × 21.84 - 25.48(24.66±2.03) (23.66±1.97)	3.5128.0735.0933.33	64
3.	Manimahesh Lake		24.52 × 25.13	100	26
4.	Jalori Pass		36.40-40.04 × 36.40(37.44±1.71)32.76 × 25.48-32.76(31.08±2.83)25.48 × 25.4821.84 × 21.8410.92 × 10.92	1.5839.1037.5020.631.19	77
5.	Rohtang Pass		19.27-23.85 × 20.64-27.52(21.73±1.43) (25.05±1.48)16.05-16.51 × 17.89 - 18.35(16.28±0.19) (18.05±0.31)	53.9946.01	56
6.	Keylong		24.85 × 26.55	100	24
7.	Shashur		21.10 × 19.2616.13 × 16.13	86.0313.97	70

### Cytological analysis

*The diploid*(n=8) cytotype

**Figure 3A–N.**
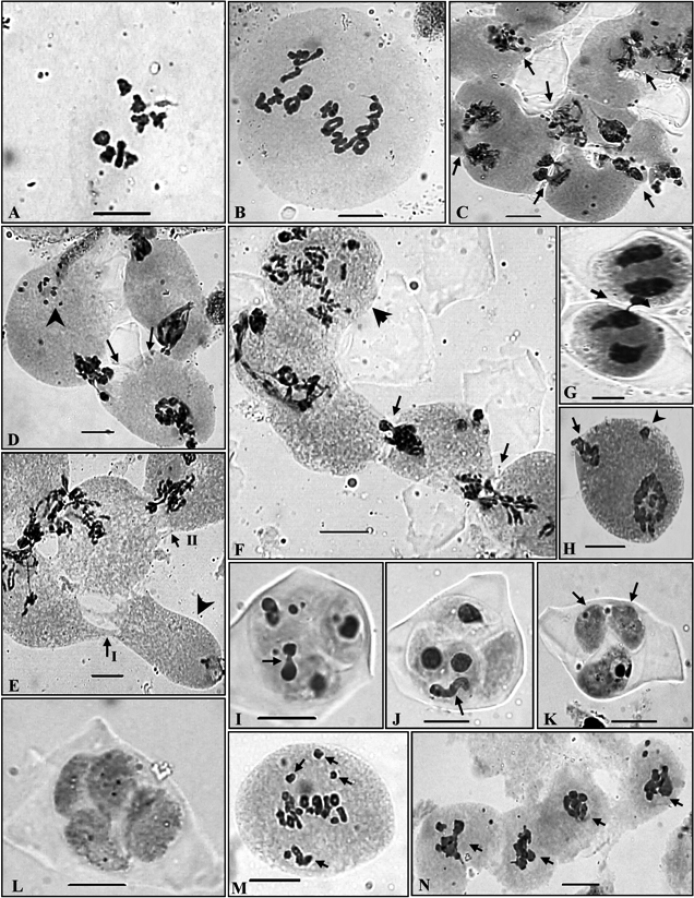
PMCs showing meiotic chromosome number and abnormal meiotic behaviour in *Ranunculus hirtellus*. **A** 2× cytotype, n=8 at MI **B** 4× cytotype, n=16 at diakinesis **C** A group of PMCs involved in the transfer of chromatin material at early prophase-I (arrowed) **D** Two PMCs (arrowed) showing simultaneous transfer of chromatin material and pycnotic chromatin material (arrowhead) **E** A group of PMCs showing narrow and broad cytoplasmic connections (arrowed) and an almost enucleated PMC (arrowhead) **F** A group of PMCs showing transfer of chromatin material (arrowed) and a hyperploid PMC (arrowhead) **G** Two PMCs involved in chromatin material transfer at TI (arrowed) **H** A PMC at TI showing broken chromatin strand at one pole (arrowed) and a laggard (arrowhead) **I, J** Microspores showing transfer of chromatin material within the sporads (arrowed) **K** Two empty microspores (arrowed) without any chromatin material in a sporad **L** Completely empty microspores in a sporad **M** Out of metaphase plate bivalents (arrowed) **N** A group of PMCs showing chromatin stickiness (arrowed).

Only one population growing on the moist alpine slopes of Gauri Kund (3930 m) in the Manimahesh hills (Chamba district) existed at diploid level (based on x=8) as confirmed from the presence of 8 medium sized bivalents in the PMCs at MI (Fig. 3, A). These bivalents showed regular segregation during AI. Further meiotic course was also regular resulting into normal tetrad formation, nearly cent per cent pollen fertility and uniform sized pollen grains.

*The tetraploid* (n=16) cytotype

**Figure 4A–N.**
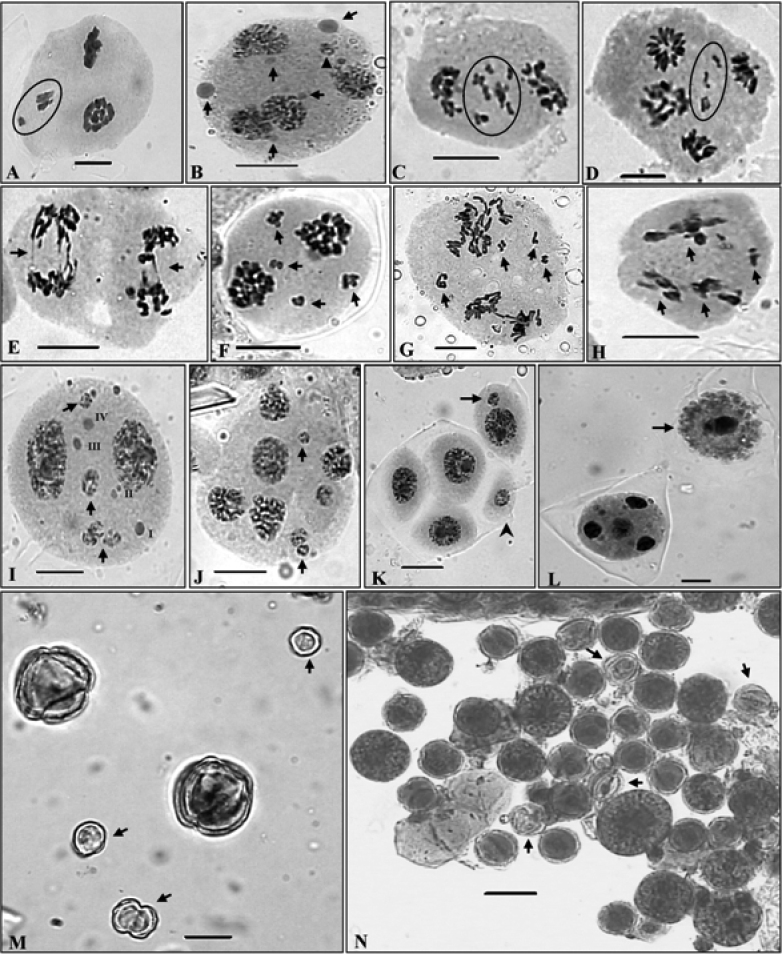
Meiotic abnormalities and pollen grains. **A** A PMC showing extra chromatin material (encircled) **B** A PMC showing unequal sized supernumerary nucleoli (arrowed) and micronuclei (arrowhead) **C, D** Laggards at late AI/II (encircled) **E** Chromatin bridges at AII (arrowed) **F–H** PMCs showing disoriented chromosomes in multiple groups (arrowed) **I** A PMC with micronuclei (arrowed) and supernumerary nucleoli (I–IV) **J** A polyad with micronuclei (arrowed) **K** Sporad with included micronuclei in microspore (arrowed) and a microcyte (arrowhead) **L** A monad (arrowed) and a normal PMC with four haploid nuclei at TII **M** Very small sized sterile/unstained (arrowed) and large lightly stained pollen grains **N** Stained apparently fertile heterogeneous sized and sterile/unstained (arrowed) pollen grains. Scale bars = 10 μm, except micrograph N=20 μm

The tetraploid cytotype has been found to be more common as confirmed from the presence of meiotic chromosome number of n=16 in six out of the seven populations scored presently from the different localities in the Himalayas. These tetraploid individuals in all the populations unequivocally showed the presence of 16 bivalents in the PMCs (Fig. 3, B). In spite of normal bivalent/s formation and their equal distribution during anaphases, PMCs showed various meiotic abnormalities which include PMCs involved in chromatin transfer at different stages (Fig. 3, C-L), out of plate bivalent/s (Fig. 3, M), chromatin stickiness (Fig. 3, N), pycnotic chromatin (Fig. 3, D), extra chromatin in PMCs (Fig. 4, A), supernumerary nucleoli (Fig. 4, B, I), laggards and chromatin bridges (Fig. 4, C-E), micronuclei (Fig. 4, I, J) and disoriented chromatin material at anaphases/telophases (Fig. 4, F-H). Consequent to these meiotic abnormalities, abnormal sporads (Fig. 4, J-L) were produced which lead to varying percentages of pollen sterility (24 - 77 %) and pollen grains of heterogeneous sizes (Fig. 4, M & N). The data on cytomixis, meiotic course, microsporogenesis and pollen sterility and pollen size in each population of the tetraploid cytotype are provided in the Tables 3, 4.

**Table 4.** Cytomixis, meiotic course and microsporogenesis in the 4× cytotype of *Ranunculus hirtellus*. PMC = pollen mother cell; M-I = metaphase-I; P-I = Prophase-I; AI/TI= anaphase-I/telophase-I; AII/TII = anaphase-II/telophase-II;

Populations	Cytomixis			Meiotic course				Microsporogenesis
	% age of PMCs involved	No. of PMCs involved	Meiotic stage/s	PMCs with chromosome stickiness (%)	PMCs with laggards at AI/TI, AII/TII (%)	PMCs with bridges at AI/TI, AII /TII (%)	PMCs with unoriented chromatin material (%)	Abnormal sporads (tetrads and polyads with and without micronuclei)
Dhancho	5.33	2-3	M-I	18.10	35.90	1.93	---	---
Manimahesh Lake	15.95	2-3	P-I	---	6.83	---	---	---
Jalori Pass	26.40	2-5	P-I, M-I	---	11.40	2.50	---	---
Rohtang Pass	22.85	2-4	M-I	---	53.85	---	30.80	---
Keylong	26.47	2-3	M-I	---	5.03	---	---	15.55
Shashur	26.17	2-4	P-I, M-I, T-I	---	26.53	---	---	44.49

### Characteristics of meiotic abnormalities

Cytomixis involving inter PMC transfer of chromatin material was observed only during the meiotic stages of meiosis-I (Table 4). The chromatin transfer which occurred through narrow as well as broad cytomictic channels among 2–5 proximate PMCs leads to the formation of PMCs with extra chromatin material (Fig. 3, C-H & 4, A). Transfer of chromatin material among PMCs was observed to be both unidirectional as well as bidirectional forming 1–2 chromatin strands. Hypo-, hyperploid and enucleated PMCs were resulted due to partial and complete transfer of chromatin material (Fig. 3, E, F). Interestingly in few instances transfer of chromatin material occurred simultaneously from two PMCs to a single recipient PMC (Fig. 3, D). In some cases remnants of chromatin strands which existed between proximate PMCs during cytomixis were seen as broken chromatin strands (Fig. 3, H). Out of plate bivalent/s at MI was also noticed in a few PMCs (Fig. 3, M). Chromatin stickiness mostly existed in the meiocytes at MI (Fig. 3, N, Table 4). Pycnotic chromatin formed due to chromatin stickiness was also noticed at earlier stages of prophase-I (Fig. 3, D). Some PMCs also showed the presence of supernumerary nucleoli which were of unequal sizes (Fig. 4, B, I). Other most prominent meiotic anomalies noticed were the occurrence of laggards (1–7) at anaphases/ telophases (Fig. 4, C & D, Table 3) and disorientation of chromosomes during anaphases owing to spindle irregularities (Fig. 4, F-H, Table 4). These laggards and unoriented chromatin material failed to get included at poles during telophases, and constituted micronuclei, multipolar PMCs and microcytes (small sized microspore) during sporad formation (Fig. 4, K). The number of such micronuclei in PMCs varied from 1–4 (Fig. 4, I & J). During microsporogenesis these micronuclei were observed to present either freely in the sporads along with four microspores (1–3 micronuclei as separate units) or as included in microspores (Fig. 4, K). Polyads with 1–2 micronuclei and without micronuclei were also noticed. Chromatin bridges were also observed during late AII/TII stages Fig. 4, E, Table 4). Another interesting observation in the population collected from Dhancho (3,030 m)was the occurrence of sporads with empty microspores i.e. microspores without any chromatin material in 6.40 % of cases (Fig. 3, K). Sporads with all the microspores without any chromatin material were also observed (Fig. 3, L). Transfer of chromatin within the sporad units has also been observed in some cases (Fig. 3, I & J). Besides, monads were also recorded in 2.4 % of the observed cases in the same population (Fig. 4, L). Chromatin transfer coupled with associated meiotic abnormalities and consequent abnormal microsporogenesis resulted into high pollen sterility (Table 3) and heterogeneous sized pollen grains (Fig. 4, L, Table 4).

## Discussion

### Chromosomal status, comparison of 2× and 4× cytotypes and their distributional pattern in Indian Himalayas

The present diploid (n=8) and tetraploid (n=16) chromosome counts for the species from this region of the Himalayas, explored for the first time, agree with the earlier reports from other regions of Indian Himalayas. Both the diploid, 2n=2×=16 (Mehra and Remanandan 1972, Gulmarg in Kashmir, Jammu and Kashmir) and tetraploid, 2n=4×=32 cytotype (Jee et al. 1983a, b, Kashmir, Jammu and Kashmir, Bir and Thakur 1984, Bir et al. 1987, Valley of Flowers’, Garhwal Himalayas, Uttarakhand, Kaur et al. 2010, Dalhousie hills, Chamba district, Himachal Pradesh) have been recorded from Indian Himalayas (Fig. 1). A triploid cytotype (2n=24) had also been recorded from eastern Himalayas in India by Sharma (1970) and Roy and Sharma (1971). Based on x=7 another proposed basic number for the genus *Ranunculus* (Darlington and Wylie 1955) a diploid (2n=14) from Gulmarg in Kashmir Himalayas (Koul and Gohil 1973) and tetraploid (2n=28) cytotype from other parts of Indian Himalayas (Mehra and Kaur 1963) have also been reported. It is thus apparent that the species exhibits considerable degree of variability in chromosome number and morphological characters in the Indian Himalayas. In addition to the presence of intraspecific polyploid cytotypes (2×, 3×, 4× at x=8), the species also showed the existence of diploid and tetraploid chromosomal races (2n=14, 28) at basic number of x=7.

The two intraspecific cytotypes (2×, 4× at x=8) here recorded in *Ranunculus hirtellus* from the Northwest Himalayas showed variation in vegetative and reproductive characters. Analysis of various macro- and microscopic characters in individuals with 2x and 4x cytotypes revealed that increase in ploidy level is correlated with gigantism for vegetative (plant height, radical leaf length), stomatal (density, size and index) characters and number of flowers/plant. Consequently, the individuals of 2× and 4× cytotypes of *Ranunculus hirtellus* can be distinguished from each other in the field. The 4× plants were much taller in size, and had large leaves. It is thus apparent that morphological characters in the intraspecific 2× and 4× cytotypes of *Ranunculus hirtellus* are directly correlated with the increase in ploidy level as had been reported earlier in *Capsella bursa-pastoris* (L.) Medik., 1792(Svensson 1983), *Andropogon gerardii* Vitman, 1792 (Keeler and Davis 1999), *Centaurea jacea* Linnaeus, 1753(Hardy et al. 2000), *Stevia rebaudiana* (Bertoni) Bertoni, 1905 (de Oliveira et al. 2004), *Parasenecio auriculata* (DC.) J.R. Grant, 1993 (Nakagawa 2006), *Dactylis* Linnaeus, 1753 (Amirouche and Misset 2007), *Centaurea phrygia* Linnaeus, 1753 (Koutecký 2007), *Rorippa amphibia* Linnaeus, 1753(Luttikhuizen et al. 2007), *Centaurea stoebe* Linnaeus, 1753(Španiel et al. 2008, Mráz et al. 2011), *Ranunculus parnassifolius* Linnaeus, 1753(Cires et al. 2009) andnumber of woody species (Singhal et al. 2007). There is no significant difference in the pollen grain size of the 2× cytotype and in the two populations (Keylong and Manimahesh Lake, Table 3) of the 4× cytotype where the typical pollen grains (normal) were of the same size as that of the 2× cytotype. So, the increase in the ploidy level has not affected the pollen grain size in the 4× cytotype. Different sized pollen grains in other populations of the 4× cytotypes are the product of various meiotic abnormalities (hypo- and hyperploid PMCs) and abnormal microsporogenesis (monads, polyads and sporads with microsporocytes). Generally polyploid plants are considered to have delayed flowering but in the presently studied species the flowering period among 2× and 4× cytotypes has been observed to be nearly the same. As far as the distribution of the two cytotypes are concerned, the 4× cytotype is widely distributed in different geographical areas of the Manimahesh hills, Manali hills and Lahaul Valley compared to the 2× cytotype which is restricted to the Manimahesh hills. Furthermore, on the basis of overall information gathered from the works of other Indian workers from Himalayas it becomes more clear that the 4× cytotype is widely distributed in the Kashmir Himalayas (Jee et al. 1983a, b), Garhwal Himalayas (Bir and Thakur 1984, Bir et al. 1987) and other regions of the Indian Himalayas. On the other hand, the 2× cytotype has been recorded earlier only from Gulmarg (Kashmir Himalaya) by Mehra and Remanandan (1972) and Koul and Gohil (1973).

### Meiotic course

The male meiotic course in the meiocytes was perfectly normal in the diploid cytotype resulting into cent percent pollen fertility. However, all the individuals of the 4× cytotype showed the phenomenon of cytomixis involving chromatin transfer among proximate PMCs and various other associated meiotic abnormalities. Consequently very high pollen sterility and fertile pollen grains of two heterogeneous sizes were resulted. The phenomenon of cytomixis is reported here for the first time in the species.

### Cytomixis in the PMCs of tetraploid cytotype

Transfer of chromatin material between the adjacent PMCs occurred through cytomictic channels and these cytoplasmic channels originating from the pre-existing connections of plasmodesmata formed within the anther tissues. As meiosis progress these connections get obstructed by the callose plugs. However, in some cases they may exist till the later stages of meiosis and their size may increase to form conspicuous inter-PMC cytomictic channels through which transfer of chromatin or chromosomes may take place (Falistocco et al. 1995, Haroun 1995, Singhal and Kumar 2008a, b, 2010, Kumar et al. in press, Shabrangi et al. 2010, Mursalimov and Deineko 2011). Chromatin transfer was reported for the first time in gymnosperms by Arnoldy (1900) and subsequently by Koernicke (1901). However, it was Gates who coined the term cytomixis after eleven years in 1911. Since that time it has been reported in a large number of plants. Occurrence of cytomixis only in the tetraploid cytotype and not in the diploid individuals in *Ranunculus hirtellus* confirms the view of other workers that the phenomenon is more prevalent in polyploids than their diploid counterparts (Kamra 1960, Semyarkhina and Kuptsou 1974, Basavaiah and Murthy 1987, Sheidai and Attaei 2005).

Chromatin transfer occurred through variable sized cytoplasmic channels forming 1–2 chromatin strands involving 2–5 PMCs and the percentage of meiocytes involved in cytomixis ranged between 5.53–26.47%. The chromatin material transfer was observed only during the early stages of the meiosis-I, which confirmed the view of other workers that earlier stages of meiosis-I are more favourable for cytomixis (Maheshwari 1950, Kundu and Sharma 1988, Sen and Bhattacharya 1988, Haroun 1995, Singhal and Kumar 2010). Some of the PMCs were also directly fused to facilitate the chromatin transfer. In some cases, cytomixis may lead to the migration of the whole chromatin material among the neighbouring meiocytes and lead to the formation of unreduced gametes. Hypo-, hyperploid and enucleated meiocytes observed at different meiotic stages were the result of partial or complete chromatin transfer between meiocytes. And the products of such PMCs in these individuals yield variable sized apparently fertile and sterile pollen grains. Various workers who considered cytomixis to be of considerable significance, the most probable consequence of cytomixis is the formation of hypo-, hyperploid and enucleated PMCs, aberrant microspore tetrads and pollen sterility (Haroun 1995, Caetano-Pereira and Pagliarini 1997, Malallah and Attia 2003, Haroun et al.2004, Singhal et al. 2007, 2008, 2009a, b, 2010, Kumar and Singhal 2008, Singhal and Kumar 2008a, b).

Another rare and interesting observation recorded during the meiotic course of *Ranunculus hirtellus* was the occurrence of sporads with empty microspores. In some cases sporads were devoid of any chromatin material. One of the possible explanations for the presence of empty microspores in a sporad is the transfer of chromatin within the sporad. Completely empty sporads might have resulted due to the transfer of chromatin between the units of two different sporads. To the best of our knowledge this is the first report of the occurrence of empty microspore units in sporads which were devoid of chromatin material due to complete transfer of chromatin material among microspores of sporads.

### Other meiotic abnormalities

The other most frequently observed meiotic abnormalities included laggards and bridges at anaphase/telophase, chromatin stickiness, pycnotic chromatin and aberrant spindle activity in the PMCs which possibly have been induced by cytomixis (Kumar and Singhal 2008, Singhal and Kumar 2008a, b, 2010, Kumar et al. 2008a, 2010). These laggards when failing to get included in telophase nuclei resulted in the formation of micronuclei at late telophase and sporad stage. The presence of extra chromatin material in the recipient meiocytes due to chromatin transfer also contributed to the formation of micronuclei at late telophase and sporad stage. Chromosome stickiness also resulted in the formation of pycnotic chromatin at earlier stages of prophase-I and chromatin bridges at anaphase/telophase. The normal functioning of spindle apparatus is crucial for chromosome alignment during metaphase and correct segregation of chromosomes to poles (Shabrangi et al. 2010). Disturbed spindle apparatus orientation may have resulted in scattered and disoriented chromosomes in the meiocytes. Spindle irregularity in *Ranunculus hirtellus* resulted in unoriented chromatin material at anaphase/telophase which led to the formation of laggards, and these laggards subsequently failed to be included in the telophase haploid nuclei and yielded micronuclei at late telophases and in the sporads.

The phenomenon of cytomixis has been reported a large number of angiospermic plants, and many workers consider cytomixis to be of considerable evolutionary significance (Falistocco et al. 1995, Morikawa and Leggett 1996, Malallah and Attia 2003, Singhal and Kumar 2010). But so far no consensus regarding its fate, cause and importance has been developed due to different opinions and explanations. Some of the possible causes and explanations put forth by the earlier workers include the effect of fixation (Haroun 1995), pathological changes (Morisset 1978), physiological control (Bahl and Tyagi 1988), chemical and herbicides (Haroun 1995), environmental stress and pollution ( Haroun et al. 2004), temperature (Kumar and Tripathi 2008), stress factors and genetic control (Malallah and Attia 2003), pressure difference (Morisset 1978) and clumped chromatin bridges during premeiotic anaphase (Mendes and Rijo 1951). The impact of cytomixis and chromatin transfer in inducing various meiotic abnormalities in *Ranunculus hirtellus* resulting into abnormal sporad formation, and some pollen malformation seem to be under some genetic factors (Singhal and Gill 1985, Bellucci et al. 2003, Haroun et al. 2004, Lattoo et al. 2006, Singhal et al. 2007, 2008, 2009a, b, 2010, in press, Kumar and Singhal 2008, Singhal and Kumar 2008a, b, 2010, Kumar et al. 2008a, b, 2010, in press, Himshikha et al. 2010) or the genetic imbalance in the 4× cytotype, high altitude and low temperature stress conditions prevailing in the cold deserts, where temperature during the months of May - July dips to below freezing, the time the plants enters the reproductive/ flowering bud stage.

### Conclusion

On the basis ofmorphological characters both the 2× and 4× cytotypes are distinguishable in the field from each other. The 4× cytotype has a wider distribution in the Indian Himalayas compared to 2× and 3× cytotypes. And the occurrence of various meiotic abnormalities in the 4× cytotype may be attributed to the genetic imbalance in the 4× cytotype, high altitude and low temperature stress conditions prevailing in the cold deserts.
